# From Mortality to Morbidity Control: A Paradigm Shift in Emphysematous Pyelonephritis Management

**DOI:** 10.7759/cureus.88901

**Published:** 2025-07-28

**Authors:** Suryaram Aravind, Punith Jain R, Velmurugan Palaniyandi, Hariharasudhan Sekar, Sriram Krishnamoorthy

**Affiliations:** 1 Urology, Sri Ramachandra Institute of Higher Education and Research, Chennai, IND; 2 Urology and Renal Transplantation, Sri Ramachandra Institute of Higher Education and Research, Chennai, IND

**Keywords:** bacterial pyelonephritis, e coli: escherichia coli, emphysematous pyelonephritis (epn), pig tail, type 2 diab

## Abstract

Emphysematous pyelonephritis (EPN) is a rare but life-threatening necrotizing infection of the kidney, typically seen in patients with poorly controlled diabetes. Once associated with high mortality and a low threshold for nephrectomy, the current approach to EPN has evolved considerably. We present the case of a 51-year-old diabetic woman with Class IIIA EPN and severe sepsis, successfully managed with a stepwise, minimally invasive strategy. Due to her cardiac comorbidities and unstable condition, she was deemed unfit for immediate internal drainage or surgery. A percutaneous nephrostomy (PCN) was performed under computed tomography (CT) guidance, which stabilized her clinically and allowed time for further intervention. Once her condition improved, a double-J stent was placed to achieve definitive drainage. She recovered completely with preserved renal function, as confirmed on follow-up imaging. This case highlights the importance of early imaging, multidisciplinary decision-making, and the expanding role of PCN not just as a bridging tool but as a key component of definitive therapy in EPN. It reinforces the emerging paradigm shift in EPN management - one that emphasizes morbidity reduction and organ preservation over radical interventions.

## Introduction

Emphysematous pyelonephritis (EPN) is an aggressive, necrotizing infection of the renal parenchyma and surrounding tissues, characterized by gas-forming organisms, such as Escherichia coli and Klebsiella pneumoniae [1, 2], and gas formation inside the kidneys. Traditionally linked with poorly controlled diabetes mellitus, EPN represents a urological emergency with rapid advancement to sepsis, multiorgan dysfunction, and high mortality if not promptly identified and treated [3].

Classically, mortality rates approached 80% in severe cases, and nephrectomy was often the only available life-saving intervention [4]. In recent years, the management paradigm has shifted from an emphasis solely on survival to a nuanced focus on reducing morbidity and preserving renal function. This evolution has driven improvements in early diagnostic imaging - especially non-contrast computed tomography (CT), which enables precise classification and staging of EPN - as well as the development of robust clinical risk stratification systems [5]. The modified National Early Warning Score (NEWS) 2 model proposed by Krishnamoorthy et al. and the Sepsis 6 scoring systems have enabled clinicians to accurately predict outcomes and tailor management strategies accordingly [6, 7]. This has facilitated early triaging and prompt escalation of care in high-risk patients while allowing for conservative, minimally invasive management in others.

The fundamentals of contemporary EPN treatment lie in early and accurate diagnosis, immediate hemodynamic stabilization, and timely drainage of the infected collecting system. Minimally invasive procedures, such as percutaneous drainage (PCD) and percutaneous nephrostomy (PCN), now serve not merely as temporizing measures but as definitive therapies in a significant proportion of patients, particularly those with Class I or II disease or those who are unfit for surgery [8, 9]. Even in more advanced cases, staged intervention beginning with percutaneous nephrostomy (PCN) followed by delayed internal drainage using double-J (DJ) stents has yielded excellent outcomes [10].

We report the case of a 51-year-old woman with uncontrolled diabetes who presented with urosepsis and radiologically confirmed Class III EPN. The timely placement of PCN facilitated source control and clinical stabilization, following which DJ stenting enabled the complete resolution of the infection and preservation of renal function. This case underscores the significance of early triage, multidisciplinary decision-making, and stepwise, minimally invasive interventions in achieving both survival and organ preservation in contemporary end-stage renal disease (ESRD) care.

## Case presentation

A 51-year-old woman with a long-standing history of poorly controlled diabetes mellitus presented to the emergency department with the acute onset of fever, dysuria, and severe left flank pain. She appeared toxic and hemodynamically unstable, consistent with clinical urosepsis. Initial evaluation revealed leukocytosis (WBC count: 26,000/mm^3^) and elevated serum creatinine (2.7 mg/dL), suggestive of acute kidney injury (Table 1).

**Table 1:** Baseline haematological, biochemical, and microbiological investigations All values are from the initial evaluation prior to definitive surgical intervention. Reference ranges are based on institutional laboratory standards. HPF: High-power field; HbA1c: Glycated haemoglobin; CFU: Colony forming units; No Growth / Sterile: no microbial growth observed after incubation.

Parameters	Patient Value	Units	Reference Range
Haemoglobin	8.7	g/dl	13.0 – 17.0
Total Leucocyte Count	26000	cells/mm^3^	4,000 – 11,000
Platelet Count	1,35,000	cells/mm^3^	150,000 – 450,000
Random Blood Sugar	264	mg/dl	< 140
HbA1c	9.1	%	< 5.7
Blood Urea Nitrogen	56	mg/dl	6 – 20
Serum Creatinine	2.7	mg/dl	0.7 – 1.2
Sodium	126	mEq/L	136 – 145
Potassium	5.8	mEq/L	3.5 – 5.1
Chloride	94	mEq/L	98 – 107
Bicarbonate	15	mEq/L	22 – 29
Lactate	2.2	mmol/L	0.20-1.80
Urine Pus Cells	100 – 150	/HPF	0 – 5
Gram Stain (Urine)	Gram-negative bacilli	-	No organisms / Sterile
Urine Culture	E. coli	100000	No Growth / Sterile
Blood Culture	No Growth	-	No Growth / Sterile
Pus Culture	E. coli	100000	No Growth / Sterile
ANTIBIOTICS	SENSITIVITY	-	ORGANISM ISOLATED E. coli >100000 CFU/mL
Amikacin	Susceptible	-	-
﻿﻿﻿Amoxyclav	Susceptible	-	-
﻿﻿﻿Cefoperazone with Sulbactam	Susceptible	-	-
﻿﻿Cefepime	Susceptible	-	-
﻿﻿﻿Ceftriaxone	﻿﻿﻿Resistant	-	-
﻿﻿﻿Cefuroxime	﻿﻿﻿Resistant	-	-
﻿﻿﻿Ciprofloxacin	﻿﻿﻿Intermediate	-	-
﻿﻿Cotrimoxazole	Susceptible	-	-
﻿﻿﻿Fosfomycin	Susceptible	-	-
﻿﻿﻿Gentamicin	Susceptible	-	-
﻿﻿﻿﻿Nitrofurantoin	Susceptible	-	-
Piperacillin with Tazobactum	Susceptible	-	-
Meropenem	Susceptible	-	-

**Figure 1:**
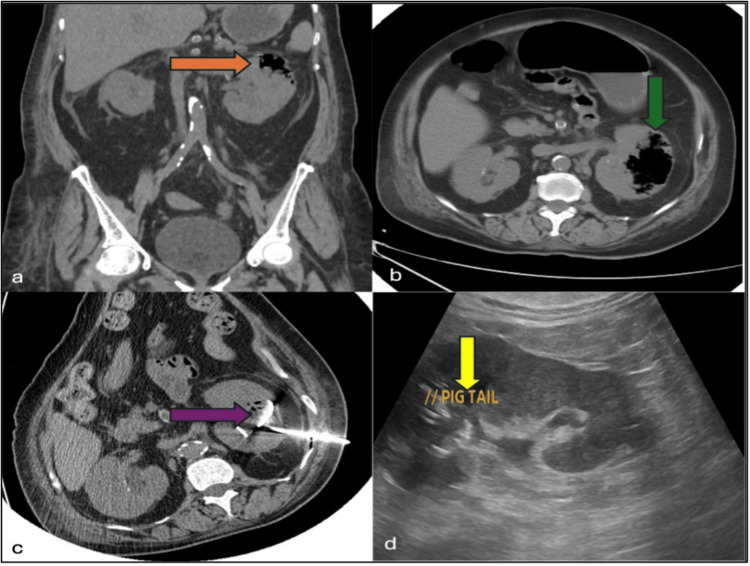
Multimodality Imaging of Emphysematous Pyelonephritis Demonstrating Gas-Forming Infection and Percutaneous Drainage. (a, b) Coronal and axial CT imaging showing the gas in the renal parenchyma. (c, d) CT and Ultrasonogram images depicting the placement of a percutaneous nephrostomy.

Ultrasound of the abdomen showed left-sided moderate hydronephrosis. Given the clinical suspicion of a severe upper urinary tract infection, an urgent non-contrast computed tomography (CT) scan was performed in the emergency setting (Figure 1).

The scan confirmed Class IIIA emphysematous pyelonephritis (EPN), which showed 30% destruction of renal parenchyma, with gas extending into the renal parenchyma and perinephric space but confined within Gerota’s fascia. No evidence of calculus was noted. These findings, coupled with her clinical deterioration, necessitated immediate intervention.

The patient was deemed high-risk for anesthesia due to underlying congestive heart failure and ongoing sepsis. Given these constraints, CT-guided percutaneous nephrostomy (PCN) was promptly performed as a life-saving decompression strategy by a teamed approach with a urologist and interventional radiologist. A 12 Fr pigtail catheter was inserted into the renal pelvis, yielding approximately 50 mL of purulent material. After culture and sensitivity, the appropriate intravenous broad-spectrum antibiotic, Meropenem, was administered, and the patient was closely monitored in the intensive care unit. Over the following days, there was a marked clinical improvement - her fever subsided, leukocyte counts normalized, and renal function stabilized. Serial imaging demonstrated a reduction in perinephric gas and fluid collections. Once cardiac parameters improved, internal drainage with a double-J (DJ) stent was undertaken to facilitate definitive urinary diversion. The nephrostomy catheter was subsequently removed.

**Figure 2:**
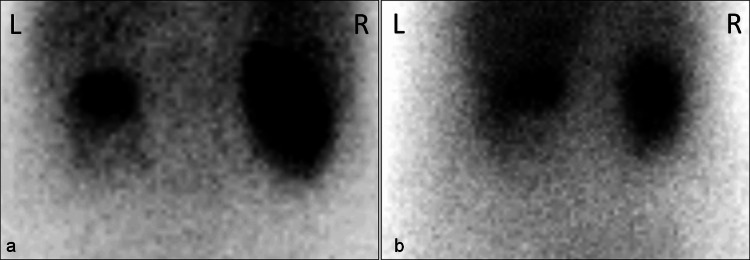
Post-Treatment Renal Scintigraphy Demonstrating Preserved Cortical Function in the Affected Kidney

Urine culture revealed the presence of Escherichia coli, which was sensitive to the ongoing antimicrobial regimen. She was discharged in stable condition. A follow-up dimercaptosuccinic acid (DMSA) renal scan done after six weeks revealed residual scarring and a relative function of 23% in the affected kidney, highlighting the renal impact of the infection despite salvage (Figure 2).

This case underscores the critical importance of early imaging, prompt intervention, and a staged, minimally invasive approach in managing high-risk EPN. Timely source control through PCN not only reversed impending septic shock but also enabled renal preservation, avoiding the need for nephrectomy in a critically ill patient.

## Discussion

Emphysematous pyelonephritis (EPN) represents one of the most fulminant forms of upper urinary tract infection and is frequently encountered as a urological emergency in patients with uncontrolled diabetes mellitus (DM) [6]. The combination of DM and upper tract obstruction has historically conferred a dismal prognosis, with mortality once reported to be as high as 71%. Past three decades, the rapid advancements in radiological diagnosis, early recognition of prognostic markers, and the adoption of minimally invasive strategies have significantly improved outcomes, with recent mortality rates declining to 3-30% [11,12].

A substantial contribution to this shift has come from clinical scoring systems that enable risk-adapted treatment. Jain et al. highlighted that thrombocytopenia, acute kidney injury, altered mental status, and shock at presentation were independent predictors of poor outcome [13]. Their proposed prognostic score enables clinicians to stratify patients into low-, intermediate-, and high-risk groups, thereby tailoring management from the outset. Similarly, Fatima et al. developed a risk prediction model integrating radiological class with clinical and laboratory parameters to identify patients who would benefit from conservative management over nephrectomy [14].

These frameworks have empowered clinicians to shift from a mortality-centered to a morbidity-conscious management philosophy. Nephrectomy, once the gold standard in unstable or non-responding patients, has increasingly been reserved for select refractory cases. Multiple studies, including those by Kapoor et al. [3] and Somani et al. [11, 15], have demonstrated that percutaneous drainage, when combined with appropriate antimicrobial therapy, can achieve comparable outcomes to nephrectomy in Class I and II disease.

Our patient, a 51-year-old postmenopausal woman with long-standing diabetes and heart failure, presented with severe urosepsis with clinical and radiological findings consistent with Class IIIA EPN. She exhibited several adverse prognostic indicators, including leukocytosis, renal dysfunction, and systemic inflammatory response. Based on traditional paradigms, immediate nephrectomy might have been considered a viable option. However, her underlying cardiac dysfunction and elevated perioperative risk precluded general anaesthesia, necessitating an alternative approach.

A CT-guided PCN was performed as the first-line intervention. A 12 Fr pigtail catheter was inserted into the renal pelvis, draining 50 mL of purulent fluid. This intervention allowed immediate decompression of the collecting system and provided a sample for culture and sensitivity testing. Empirical broad-spectrum intravenous antibiotics were initiated and later adjusted based on the growth of Escherichia coli. Clinical improvement was evident over the subsequent days, with resolution of fever, normalization of the leukocyte count, and stabilization of serum creatinine levels.

Attempts at internal drainage via DJ stenting under local anaesthesia were deferred during the acute phase due to suspected obstruction by necrosed renal papilla. Manipulation during this period would have risked precipitating bacteremia or worsening sepsis. Once the patient’s cardiac status improved and the inflammatory response abated, internal drainage with a DJ stent was successfully performed under monitored anaesthesia. The nephrostomy catheter was subsequently removed after confirmation of adequate drainage and clinical resolution.

This staged, minimally invasive approach - PCN followed by delayed DJ stenting - stabilized the patient’s systemic condition and preserved renal function, as confirmed by the follow-up DMSA scan. Although residual scarring and reduced function were noted on the affected side, nephron-sparing management was successfully achieved.

EPN often mimics severe pyelonephritis or sepsis, making early differentiation difficult. Laboratory abnormalities such as leukocytosis, elevated serum creatinine, metabolic acidosis, and thrombocytopenia are common but nonspecific. Thus, early imaging, particularly non-contrast CT, remains indispensable for diagnosis and radiological staging. In our case, timely imaging was crucial in recognizing the extent of parenchymal involvement and guiding urgent intervention.

This case reiterates the evolving role of PCN, not as a temporizing measure, but as a cornerstone in the conservative management of EPN, especially in unstable patients or those unfit for surgery [16]. The benefits of PCN include immediate decompression, source control, avoidance of general anaesthesia, and a bridge to definitive internal drainage. Growing evidence supports renal salvage rates of 80-90% with PCN in appropriately selected cases [17,18].

In summary, the management of EPN has shifted significantly from radical surgical intervention to risk-adapted, organ-preserving therapy guided by validated clinical and radiological prognostic markers. The integration of predictive scoring systems enables early triaging and individualized care. Our case reinforces that a stepwise, patient-centred approach involving PCN, sepsis control, and delayed stenting can lead to favourable outcomes, even in high-risk scenarios.

To our knowledge, this is the first case report to document a successful, staged organ-preserving strategy for Class IIIA EPN in a hemodynamically unstable patient with significant cardiac comorbidity, where initial decompression with CT-guided percutaneous nephrostomy (PCN) followed by delayed internal drainage via double-J stenting under monitored anesthesia led to complete clinical recovery and renal salvage - despite the absence of surgical intervention or general anesthesia throughout the course.

## Conclusions

This case emphasizes the importance of early detection and radiological diagnosis of emphysematous pyelonephritis. Non-contrast computed tomography of the kidneys, ureters, and bladder (CT KUB) was not only pivotal in diagnosing Class IIIA EPN but also in establishing the urgency and nature of treatment. Percutaneous nephrostomy is a highly effective initial strategy in patients with sepsis and multiple comorbidities, contributing to immediate decompression and infection control. It also offers the ability to administer targeted antibiotic therapy and helps in strategically delaying the risks of emergency nephrectomy or general anaesthesia. Once the patient was hemodynamically stable, the staged placement of a double-J stent enabled complete internal drainage and contributed to the preservation of renal function. This aftereffect illustrates that, even in high-risk clinical events, kidney salvage is attained when interventions are timely, minimally invasive, and tailored to the patient’s physiological needs. The evolving approach to EPN management increasingly prioritizes not only survival but also the reduction of morbidity and the preservation of renal function. Outcomes are further optimized when treatment is guided by imaging, adapted to individual risk profiles, and delivered through a coordinated, multidisciplinary framework.
